# Fe(III) and Cu(II) Complexes of Chlorogenic Acid: Spectroscopic, Thermal, Anti-/Pro-Oxidant, and Cytotoxic Studies

**DOI:** 10.3390/ma15196832

**Published:** 2022-10-01

**Authors:** Monika Kalinowska, Kamila Gryko, Ewelina Gołębiewska, Grzegorz Świderski, Hanna Lewandowska, Marek Pruszyński, Małgorzata Zawadzka, Maciej Kozłowski, Justyna Sienkiewicz-Gromiuk, Włodzimierz Lewandowski

**Affiliations:** 1 Department of Chemistry, Biology and Biotechnology, Institute of Environmental Engineering and Energetics, Faculty of Civil Engineering and Environmental Sciences, Białystok University of Technology, Wiejska 45E Street, 15-351 Bialystok, Poland; 2 Institute of Nuclear Chemistry and Technology, 16 Dorodna Street, 03-195 Warsaw, Poland; 3 NOMATEN Centre of Excellence, National Centre of Nuclear Research, 7 Andrzeja Soltana Street, 05-400 Otwock, Poland; 4 Department of General and Coordination Chemistry and Crystallography, Institute of Chemical Sciences, Faculty of Chemistry, Maria Curie-Sklodowska University, Maria Curie-Sklodowska Sq. 2, 20-031 Lublin, Poland

**Keywords:** phenolic compounds, metal complexes, chlorogenic acid, caffeoylquinic acid, antioxidant activity

## Abstract

Complexes of chlorogenic acid (5-CQA) with copper(II) and iron(III) were synthesized in a solid state and examined by means of FT-IR, thermogravimetric, and elemental analyses. The molar stoichiometric ratios of metal:ligand for the solid forms of the complexes were established as Cu(II):L = 1:2 and Fe(III):L = 2:3 (L: 5-CQA), with the possible coordination through the carboxylate group and the hydroxyl group from the catechol moiety. In an aqueous solution at pH = 7.4, the composition of the complexes was Cu(II):L = 1:1, and Fe(III):L = 1:1 and 1:2. The Cu(II) and Fe(III) complexes with 5-CQA showed lower antioxidant properties, as estimated by the spectrophotometric methods with DPPH^•^, ABTS^•+^, and HO^•^ radicals, than the ligand alone, whereas in the lipid peroxidation inhibition assay, the metal complexes revealed a higher antioxidant activity than 5-CQA. Cu(II) 5-CQA showed the highest pro-oxidant activity in the Trolox oxidation assays compared to the other studied compounds. The lipophilic parameters of the compounds were estimated using the HPLC method. 5-CQA and its complexes with Fe(III) and Cu(II) were not toxic to HaCaT cells in a tested concentration range of 0.15–1000 nM after a 24 h incubation time.

## 1. Introduction

In recent years, the interest in antioxidant compounds of natural origin has been constantly growing. A noteworthy group of chemical compounds in this area are plant phenolic acids [1]. Chlorogenic acids have recently turned out to be some of the more available and active phenolic compounds because of their antioxidant, anti-inflammatory, and anticancer properties [2]. Chlorogenic acids (caffeoylquinic acids, CQAs) are esters of one or more molecules of cinnamic acid (or its derivatives, including caffeic, ferulic and *p*-coumaric acids) and quinic acid, which belong to the hydroxycinnamic acid group [1]. Among all the isomers found in plants, 3-caffeoylquinic acid (3-CQA), 4-caffeoylquinic acid (4-CQA), and 5-caffeoylquinic acid (5-CQA, commonly called chlorogenic acid) stand out as the main ones (the structures of CQAs have been shown in previous publications) [3,4,5].

### 1.1. Physicochemical Properties

The compounds that belong to the family of chlorogenic acids exhibit very different physicochemical properties, depending on the identity, number, and position of the acyl residues esterified with quinic acid, as well as the functional groups present on the aromatic group of the acyl residues [3]. CQAs are soluble in water, but the polarity of CQAs consequently decreases with the degree of esterification, i.e., monoCQAs > diCQAs > triCQAs > tetraCQAs. The less-polar CQAs are soluble in lower alcohols or alcohol–water mixtures. They are insoluble in benzene, chloroform, and petroleum ether. The polar nature of CQA makes it relatively insoluble in the lipid matrix. Furthermore, its polyphenolic structure leads to its instability and poor penetration across the lipophilic membrane barrier, limiting its absolute bioavailability in the human organism [6]. CQAs are highly susceptible to the effects of temperature. As temperature increases, they undergo intramolecular isomerization, transesterification, and degradation more easily. CQAs are photosensitive and undergo trans–cis isomerization upon exposure to ultraviolet or visible light [3].

It has been demonstrated that 5-CQA not only isomerizes to 3-O-caffeoylquinic acid and 4-O-caffeoylquinic acid, but also undergoes other transformations such as esterification and reactions with water, i.e., hydrolysis and/or the addition of a water molecule to the double bond. These processes occur not only in CQA solutions, but also during their isolation from plant materials and can lead to extracts with a lower content of biologically active phenolic compounds [3].

### 1.2. Biological Properties

**Figure 1:**
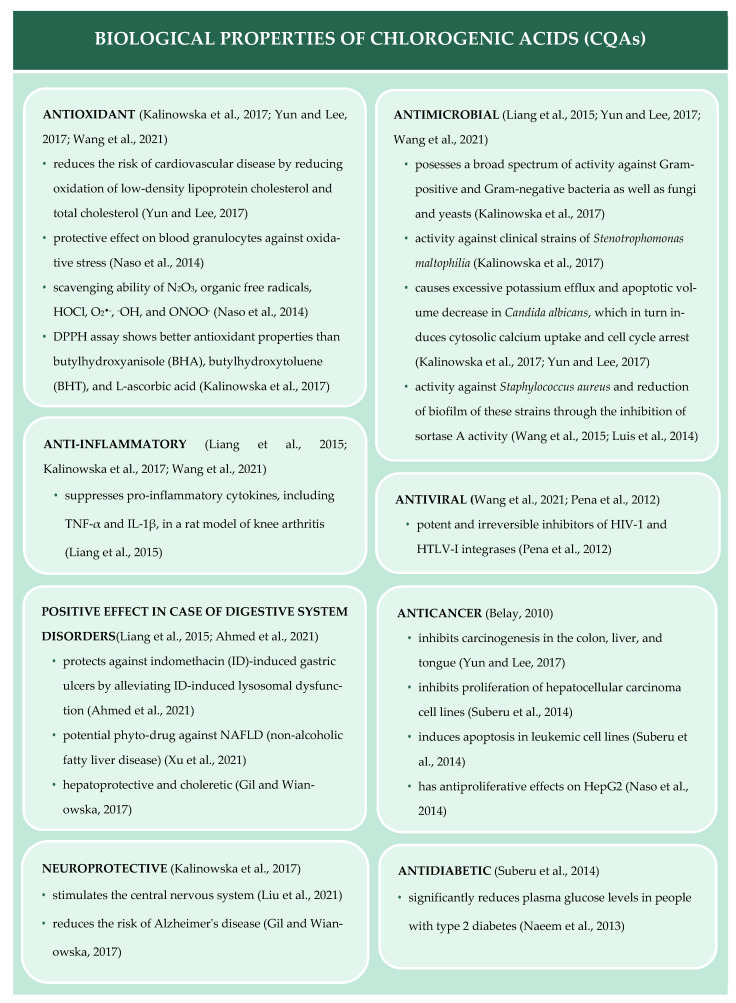
Biological properties of chlorogenic acids (CQAs) [2,4,5,11,12,13,14,15,16,17,18,19,25,26,27].

Chlorogenic acids exhibit a wide range of health-promoting properties (Figure 1), which makes them an attractive food additive or drug. Chlorogenic acids exhibit antioxidant [4,7,8,9,10], anti-inflammatory [2,4,7], antibacterial [2,4,7,8,11,12], antiviral [7,13], anticancer [8,9,14,15], neuroprotective [3,4,16], and antidiabetic [14,17] effects, as well as positive effects against gastrointestinal diseases [2,3,18,19]. In addition, they can increase the number of white blood cells, lower blood pressure, and stimulate the central nervous system [16].

In vitro and in vivo data indicate that 5-CQA has antioxidant activity and can alleviate oxidative stress in various disease models [2]. According to their structure, phenols can eliminate radicals directly, through a peroxidase reaction or by forming chelates with metal ions, thus preventing Fenton-type reactions [20]. Despite their significant antioxidant properties, CQAs are also characterized by pro-oxidant properties, which depend on their concentration, the occurrence of transition metal ions, and environmental conditions (presence of oxygen molecules, high pH value) [7,19,20,21,22]. Moreover, the mechanism of the pro-oxidant action of CQAs can be based on the ability to produce reactive oxygen species (ROS), i.e., hydrogen peroxide H_2_O_2_, superoxide radicals ROO^•−^, and hydroxyl radicals HO^•−^ formed in the Fenton reaction during the reduction of Fe(III) to Fe(II) [23]. The excessiveness of ROS in an organism can contribute to irreversible damage to the proteins, lipids, and nucleic acids present in the cells [24].

In addition, CQAs exhibit broad-spectrum activity against Gram-positive and Gram-negative bacteria as well as fungi and yeasts [5]. 5-CQA shows activity against *Stenotrophomonas maltophilia*, *Candida albicans* (MIC = 80 μg/mL) [5,12], and *Staphylococcus aureus* (MIC = 500 µg/mL) [5,13,25]. 

### 1.3. Bioavailability and Metabolism

The pharmacokinetics of CQAs have been studied. In an experimental model involving humans and animals, CQAs and their metabolites were noted in the blood. One third of ingested CQAs in beverages and food are absorbed in the small intestine, which can be measured by high-performance liquid chromatography as 5-CQA, 4-CQA, and 3-CQA present in the plasma. The remaining two-thirds enters the large intestine, where the phenolic acid is further metabolized by the gastrointestinal microflora and then absorbed [26].

Chlorogenic acids (acyl-quinic acids), as with other polyphenols, show low bioavailability due to several factors: interactions with the food matrix, and metabolic processes in the liver (phase I and II metabolism), intestines, and microflora. On the other hand, the biological activity of phenolic compounds may be mediated by their metabolites, which are produced in vivo, and recent studies have confirmed that these molecules may have antioxidant and anti-inflammatory properties. For example, unabsorbed in the small intestine, dietary 5-CQAs are hydrolyzed into caffeic and quinic acid and both are then metabolized by the colonic microflora to a series of lower-molecular-weight metabolites such as ferulic acid, isoferulic acid, *p*-coumaric acid, and gallic acid, which are mainly absorbed in the colon. The remaining metabolites enter the bloodstream and are absorbed or further metabolized (e.g., to vanillic or protocatechuic acid) in the liver [28,29].

Various ways to increase the bioavailability of CQAs have been investigated, including metal complexation, nanoformulation, and the synthesis of sulfonate derivatives. Zhang et al. studied the interactions of chlorogenic acid with whey proteins. CGA bound to three whey proteins, β-Lg, α-La, and BSA, mainly through hydrophobic force. The study was designed to provide information for further studies of milk proteins with polyphenols and the fabrication of whey protein-based carriers to improve the bioavailability of polyphenols [1].

### 1.4. Sources of Chlorogenic Acid

Chlorogenic acid is one of the most widely occurring polyphenols found in plants, resulting in its presence in the diet. It is present in many food products, e.g., apples, stone fruits, berries, cruciferous vegetables, celery, and potatoes [30,31,32,33,34,35,36,37] (Tables S1 and S2). However, the content of chlorogenic acid depends not only on the part of the plant, but also on its maturity and the storage conditions. Additionally, its high content can be found in processed beverages, most often in green and black tea, juices, wines, yerba mate, and coffee [4]. One of the richest dietary sources of CQA are coffee beans. The content of chlorogenic acid in green coffee beans is highly determined by their type; it may range from 6 to 12% of the coffee bean dry mass [4]. The content of this compound for many fruits and vegetables depends on their variety, the cultivation method, exposure to stresses, and storage conditions, which is very evident for apples (12–31 mg/100 mL of apple juice) [23]. However, irrespective of variety, 5-CQA is the major constituent [3].

### 1.5. Chlorogenic Acid–Metal Complexes

Phenolic compounds play a significant role in binding toxic metals in the environment [15]. CQAs have an important function in plant tolerance to toxic metals, as well as in preventing and reducing biotic and abiotic oxidative stress. The mechanism involves: (a) the secretion of chelating agents, e.g., by roots, to prevent metal uptake or (b) the production of chelating agents that bind metals in the cell wall, symplast, or vacuole [38].

**Figure 2:**
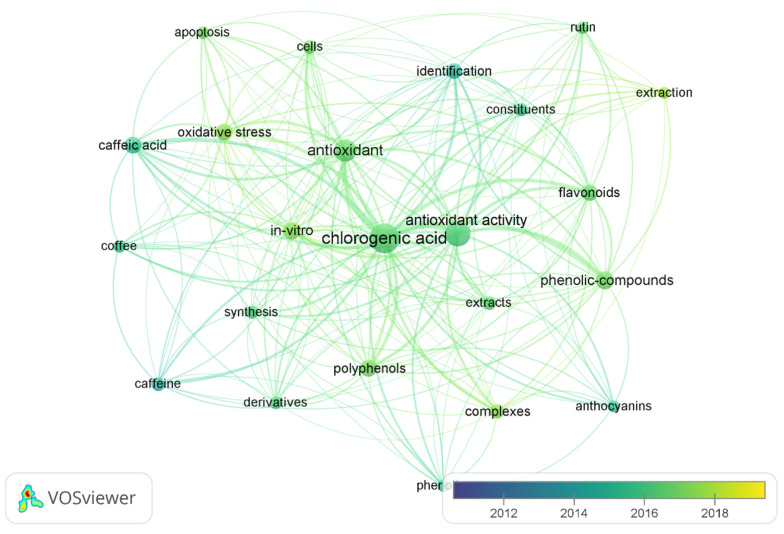
Co-occurrence of selected keywords in articles in 2010–2022 (search terms: “chlorogenic acid”, “antioxidant”), created with VOSviewer [44].

The ability of CQAs to form complexes with selected metal ions and the effect of complexation on changing their properties (including antioxidant) have been studied in recent years (Figure 2). Several publications have described the formation of complexes of CQAs with various metal ions in aqueous solutions, such as Al(III) [39], Fe(III) [40,41], Mg(II) [13], Cu(II) [42,43], or Na(I) [11]. Moreover, the ability to form CQA complexes with Pb(II) and Cu(II) and the lack of this ability for Cd(II) and Zn(II) have been demonstrated [7]. The molecular complexation of this compound with β-cyclodextrin, caffeine, and proteins has been reported to design more advanced and controlled carriers for drugs and food ingredients [15].

Complexation with metal ions can alter the antioxidant potential of chlorogenic acid. Alkali metal salts [20] and Fe(II) [40], Cu(II) [43], Ce(IV), Mg(II) [13], and Zn(II) [7] complexes of 5-CQAs showed higher antioxidant activity than the ligand itself. Kalinowska et al. [7] showed that Zn(II) 5-CQA is a better scavenger of ABTS^•+^ cationic and DPPH^•^ radicals and a better reductor of Fe(III) and Cu(II) ions compared to 5-CQA alone or even natural (L-ascorbic acid, EC_50_ = 10.32 ± 0.98 µM) and synthetic (butylated hydroxyanisol BHA, butylated hydroxytoluene BHT) antioxidants [7]. Chlorogenic acid can interact with Fe(III) to form complexes that interact with ferritin via hydrogen bonds, which promotes the rate of oxidative ion deposition and ion release from ferritin and reduces Fe(III)- and Fe(II)-induced ferritin polymerization [27]. It is important to note that antioxidants can also act as pro-oxidants depending on their concentration or the presence of certain metal cations; moreover, the stabilization of phenoxyl radicals by metal cations results in the prolongation of their lifetime [20,43]. It was shown that metals such as Al(III), Zn(II), Cd(II), Mg(II), and Ca(II) can increase the pro-oxidant activity of chlorogenic acid due to their stabilizing effect on the phenoxyl radical [43].

Therefore, in the this paper, the Fe(III) and Cu(II) complexes with 5-CQA were synthesized in a solid state and studied by means of FT-IR, thermogravimetric, and elemental analyses. The composition of the complexes in solution was estimated by means of the spectrophotometric method. The antioxidant activity of the complexes was studied by means of DPPH, ABTS, HO, lipid peroxidation inhibition, and pro-oxidant assays. The lipophilicity of the compounds was estimated by means of the HPLC method. The cytotoxic activity of Fe(III) and Cu(II) 5-CQA as well as 5-CQA toward the HaCaT cell line was studied.

## 2. Materials and Methods

Chlorogenic acid, NaOH, CuCl_2_·2H_2_O, FeCl_3_·6H_2_O, DPPH (2,2-diphenyl-1-picrylhydrazyl), ABTS (2,2′-azino-bis(3-ethylbenzothiazoline-6-sulfonic acid) diammonium salt), potassium persulfate, FeCl_2_·4H_2_O, Trolox, H_2_O_2_, horseradish peroxidase, and phosphate buffer (pH = 7) were purchased from Sigma-Aldrich and used without purification. Methanol and hydrochloric acid were purchased from Chempur (Karlsruhe, Germany).

### 2.1. Synthesis

The chlorogenates were prepared by mixing the appropriately weighed mass of chlorogenic acid (about 0.1 g weighed to 4 decimal places) with an aqueous solution of NaOH (C = 0.1 M) in a stoichiometric molar ratio of 1:1 at room temperature. Then, the aqueous solution of metal chloride (C = 0.5 M) was added to the mixture in order to obtain a molar ratio for the ligand:metal cation of 2:1 for the Cu(II) complex and 3:1 for the Fe(III) complex. Brown (Cu 5-CQA) and dark brown (Fe 5-CQA) precipitates occurred immediately. They were filtered from the solution and washed with deionized water. The precipitates were air-dried at room temperature over 48 h. The yield of the reaction was 64% and 76% in the case of Cu 5-CQA and Fe 5-CQA, respectively.

### 2.2. Thermal Analysis (TG-DCS) in Air Atmosphere and Elemental Study

The thermal behavior of the iron(III) and copper(II) chlorogenates was investigated using thermogravimetry (TG) coupled with differential scanning calorimetry (DSC). The TG-DSC measurements were conducted on a SETSYS 16/18 (Setaram, Caluire, France) thermal analyzer with dynamic air flow at a rate of 0.75 dm^3^/h. The tested samples weighing 8.149 mg and 8.770 mg were placed in alumina crucibles and heated from a temperature of 30 °C to 750 °C at a constant heating rate of 10 °C/min. The elemental analyses for the mass percentages of carbon and hydrogen were performed with Perkin–Elmer 240 equipment (PerkinElmer, Waltham, MA, USA). 

### 2.3. Spectrophotometric Determination of Cu(II) and Fe(III) 5-CQA Composition in Solution Using the Spectrophotometric Method

To determine the metal ion:ligand molar ratio in an aqueous solution of Cu(II) and Fe(III) 5-CQA, the spectrophotometric mole-ratio method was applied. The spectra in the range of 200–550 nm were recorded for solutions with a constant mole number for 5-CQA and a varied amount of Cu(II) or Fe(III) ions. The concentration of 5-CQA was 0.1 mM, while the concentration of FeCl_3_ and CuCl_2_ changed from 0 to 0.15 mM. All solutions were prepared in tris-HCl buffer (pH = 7.4; C = 50 mM).

### 2.4. IR and UV Studies

The IR spectra of 5-CQA and the Cu(II) 5-CQA and Fe(III) 5-CQA complexes in the solid state were recorded with a Cary 630 FTIR Agilent Technologies spectrometer, using the ATR technique, within the range of 400–4000 cm^−1^. The resolution was 1 cm^−1^. The UV/Vis spectra of the studied compounds at a concentration of 0.01 mM were recorded in the range of 200–550 nm in tris-HCl (pH = 7.4; C = 50 mM) using an Agilent Cary 5000 spectrophotometer (Agilent, Santa Clara, CA, USA).

### 2.5. Antioxidant Properties

#### 2.5.1. DPPH^•^

The determination of the antiradical activity of the compounds was performed by conducting a direct reaction of the DPPH^•^ radical with the tested compounds in appropriate concentrations, according to the method described in [45]. The absorbance of the samples was measured after 1 h of incubation in the dark at the wavelength λ = 516 nm. The result was the percentage of DPPH^•^ radical inhibition (%I) calculated using the formula:

$$\%I=\frac{A_{c}-A_{t}}{A_{c}}\cdot 100\%$$

where $A_{c}$ is the absorbance of the control sample and $A_{t}$ is the absorbance of the test sample.

The radical scavenging capacity was determined by the EC_50_ parameter, which is the antioxidant concentration needed to reduce the initial radical concentration by 50%. All measurements were taken for five replicates in three independent experiments.

#### 2.5.2. HO^•^

The hydroxyl radical inhibition activity was measured according to [46]. In the test sample, 0.3 mL of 8 mM FeSO_4_, 1 mL of 3 mM salicylic acid in ethanol, and 0.25 mL of 20 mM H_2_O_2_ were added to 1 mL of a 0.1 mM solution of the tested compounds. In the control sample, deionized water was added instead of H_2_O_2_, and in the blank sample, deionized water was added instead of the tested compound solution. The samples were vortexed and incubated for 30 min at 37 °C. After the incubation, 0.5 mL of deionized water was added to each sample. The samples were vortexed and the absorbance was measured immediately at 510 nm, with reference to water. The level of hydroxyl radical inhibition was calculated using the formula:

$$\%I=\left(1-\left(\frac{A_{t}^{510}-A_{c}^{510}}{A_{b}^{510}}\right)\right)\cdot 100\%$$

where $A_{t}^{510}$ is the absorbance of the test sample, $A_{c}^{510}$ is the absorbance of the control sample, and $A_{b}^{510}$ is the absorbance of the blank sample. All measurements were taken for five replicates in three independent experiments.

#### 2.5.3. ABTS^•+^

To obtain the ABTS^•+^ radical solution, ABTS (5.4 mM) and K_2_S_2_O_8_ (1.74 mM) were mixed in a 1:1 volume ratio. The mixture was then incubated in the dark for 12 h. After that, it was diluted with methanol so that, before the assay, it had an absorbance of about 0.8 at a wavelength of 734 nm [47]. A total of 1.5 mL of the diluted radical solution and 1.5 mL of 0.1 and 0.01 mM tested compound solutions were incubated in glass test tubes for 7 min. Then, the absorbance was measured at λ = 734 nm against methanol. Control samples, containing methanol instead of tested compounds solutions, were prepared in parallel. The percent of inhibition was calculated, using the same formula as for the DPPH^•^ assay. All measurements were taken for five replicates in three independent experiments.

#### 2.5.4. Lipid Peroxidation Inhibition

The lipid peroxidation inhibition capacity was tested in accordance with [48] with some modifications, by preparing linoleic acid emulsions with the addition of antioxidant at the 0.005 M concentration. From this mixture, incubated at 40 °C, 0.1 mL of the sample was taken every 24 h for 5 days. Then, methanol and 30% ammonium rhodate solution were added, and after 3 min, 0.02 M FeCl_2_ was added. The absorbance was measured immediately at the wavelength λ = 500 nm. A control sample containing no antioxidant was performed in parallel. The percent inhibition of linoleic acid peroxidation was calculated, using the formula analogous to the DPPH^•^ inhibition assay. All measurements were taken for five replicates in three independent experiments.

#### 2.5.5. Pro-Oxidant Activity

The pro-oxidant activity was measured in accordance with a method described in [49], on the basis of the compounds’ ability of Trolox oxidation. The reaction mixture contained: 0.5 mL of 0.4 mM Trolox, 0.5 mL of 0.2 mM H_2_O_2_, 0.5 mL of horseradish peroxidase in 0.05 M phosphate buffer, 50 or 25 µL of 0.1 mM tested compounds, and 0.45 or 0.475 mL of deionized water. The samples were mixed and the absorbance was measured every 10 min at λ = 272 nm. The pro-oxidant activity assay was performed in triplicate for three independent series. Calculations were made according to the following formula:

$$\%I=\frac{A_{c}-A_{t}}{A_{c}}\cdot 100\%$$

where $A_{c}$ is the absorbance of the control sample and $A_{t}$ is the absorbance of the test sample.

All measurements were taken for five replicates in three independent experiments. The absorbance was measured using an Agilent Cary 5000 spectrophotometer (Agilent, Santa Clara, CA, USA).

### 2.6. Cell Viability Assay

The influence of 5-CQA and its complexes with Fe(III) and Cu(II) on cell viability was determined by the colorimetric MTS metabolic activity assay, as described previously [50]. HaCaT is a human skin keratinocyte cell line widely used in research due to its high capacity for proliferation in vitro. It provides a reproducible model with long viability in cell culture. These cells are an excellent model of skin cells and are often used in our research alongside the Caco2, which is used as a model of the intestinal epithelial barrier. Briefly, the HaCaT human immortalized keratinocyte cells (Thermo Fisher Scientific, Inc., Waltham, MA, USA) were seeded in 96-well plates at a density of 2 × 10^3^ cells/well in a DMEM medium supplemented with 4.5 g/L of glucose, 2 mM L-glutamine, 10% fetal bovine serum, streptomycin (100 µg/mL), and penicillin (100 IU/mL). All reagents for the cell assays were from Biological Industries (Beth Haemek, Israel). After a 24 h incubation, the growth medium was replaced with one containing increasing concentrations of the tested compounds, between 0.15 and 1000 nM, and the cells were again incubated for 24 h at 37 °C. This was followed by the addition of 20 µL of 5-(3-carboxymethoxyphenyl)-2-(4,5-dimethylthiazoly)-3-(4-sulfophenyl)tetrazolium inner salt (CellTiter-96^®^ AQueous-Non-Radioactive, Promega, Mannheim, Germany). The absorbance in wells was measured at 490 nm using a micro-plate reader (Apollo 11LB913, Berthold, Bad Wildbad, Germany). The cell viability was expressed as a percentage of normalization to cells grown in medium only. All measurements were taken for three replicates in three independent experiments.

### 2.7. Lipophilicity Assay

The lipophilicity was determined using an RP-HPLC analysis using a Waters Alliance 2695 HPLC separation module (Milford, MA, USA) and a Waters 2996 photodiode array detector (Milford, MA, USA) (λ = 254 nm). The experimental methodology and the method of calculating the chromatographic lipophilicity parameter, log*k*_w_, is described in [20].

### 2.8. Statistical Analysis

To determine the statistical significance between the tested compounds, an analysis of variance (ANOVA) followed by Tukey’s test was applied. The results from three independent experiments were expressed as the mean ± standard deviation (SD) of the mean for parametric data. Significance was considered when *p* ≤ 0.05. Statistica 13.0 was used.

## 3. Results and Discussion

### 3.1. Elemental Study and Thermal Analysis of the Solid-State Samples

**Figure 3:**
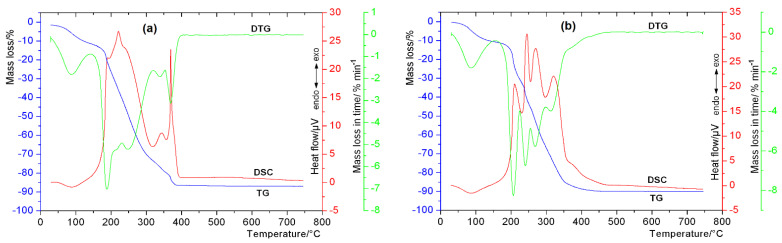
TG−DTG−DSC thermal curves for Fe(III) (**a**) and Cu(II) (**b**) chlorogenates.

Thermal behavior is an important parameter determining material properties. The TG–DTG–DSC thermal profiles of the analyzed complexes are shown in Figure 3, whereas the thermal data are gathered in Table 1. Additionally, the elemental analysis results obtained for the Cu(II) and Fe(III) 5-CQAs complexes in the solid state are presented in Table 2.

The results showed that Cu(II) formed a complex with 5-CQA with a molar ratio of 1:2 (metal:ligand), while for Fe(III) 5-CQA, the molar ratio was 2:3 (metal:ligand). The thermal decomposition of the complexes in question can be divided into two main stages. The first stage was connected only with the endothermic dehydration process, whereas the second one was related to the strong exothermic decomposition of anhydrous complexes. The presence of a weak endothermic peak seen on the DSC curves at 88 and 91 °C for the Cu(II) and Fe(III) complexes, respectively, confirmed the hydrated nature of both analyzed materials. The identified mass loss between 30 °C and 150 °C for Cu 5-CQA (10.37%) was almost the same as the calculated theoretical value (10.46%), which corresponded to the separation of five water molecules. The Fe(III) complex also contained water as a solvent, but in a different amount than its predecessor. The experimental value of the breakdown of the water molecules in the range of 30 to 140 °C (10.69%) was near the theoretical one (10.95%), which confirmed the presence of eight solvent molecules in the case of Fe(III) 5-CQA. The second key stage of the thermal decomposition was attributed to the exothermic degradation of the organic 5-CQA ligand. The loss of the organic part of the complexes occurred in several steps, as evidenced by the presence of a number of exothermic jumps on the registered DSC curves. The Cu(II) complex showed a slightly higher thermal stability than the Fe(III) compound (the thermal decomposition of the Cu(II) complex started at 150 °C, whereas the Fe(III) complex began to decompose at 140 °C). The final products of the thermal decomposition were the corresponding metal oxides: brick-red Fe_2_O_3_ and black CuO.

### 3.2. FT-IR Studies of the Solid-State Samples

**Figure 4:**
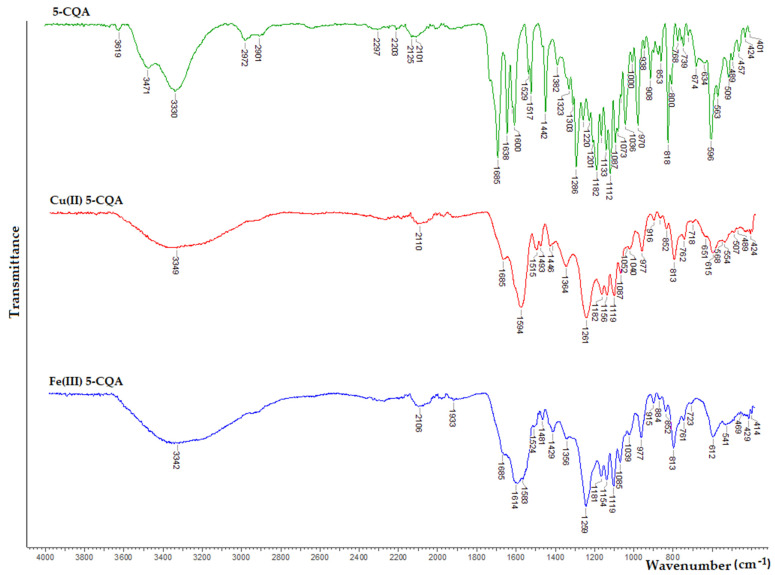
FT-IR spectra of chlorogenic acid and its complexes.

The FT-IR spectra of the Cu(II) and Fe(III) chlorogenates are shown in Figure 4, and the assignment of the selected bands are gathered in Table S3. The assignment was based on our previous publications [7,10] concerning chlorogenic acid and a zinc(II) complex with chlorogenic acid.

In the FT-IR spectra of the Cu(II) and Fe(III) complexes, the bands related to the vibrations of the carboxylate anion appeared, compared to the absence of these bands in the spectra of the ligand. The asymmetric ν_as_(COO^−^) and symmetric ν_s_(COO^−^) stretching vibrations of the carboxylate anion occurred at 1594 and 1364 cm^−1^ in the spectra of Cu(II) 5-CQA and at 1614 and 1583 cm^−1^ and 1364 and 1356 cm^−1^ in the spectra of Fe(III) 5-CQA. In the spectra of the metal complexes, there were no bands derived from the stretching vibrations of the C=O carbonyl group, which was present in the spectrum of the acid at 1725 cm^−1^. Moreover, in the spectra of the complexes, the bands assigned to the bending vibrations in-plane and out-of-plane of the carboxylate anion occurred at 813 cm^−1^ as well as 615 and 612 cm^−1^ in the spectra of the Cu(II) and Fe(III) complexes, respectively. Moreover, significant changes in the position of the band originating from the vibrations of the catechol group were observed. In the spectrum of the acid, this band is located at 1286 cm^−1^, while in the spectrum of the Cu(II) and Fe(III) complexes, it is located at 1261 and 1259 cm^−1^, respectively. The coordination through the carboxylate group probably affected the whole structure of the ligand, including the catechol moiety. On the other hand, these metal ions may form oligomeric structures with chlorogenic acid, similar to the zinc complex [7]. Some of the other bands present in the FT-IR spectra of the 5-CQA complexes were slightly shifted or disappeared compared with the spectra of 5-CQA. This means that the metal ions affected the structure of the quinic and caffeic acid moieties.

### 3.3. Determination of the Composition of Cu(II) and Fe(III) 5-CQAs in Solution

**Figure 5:**
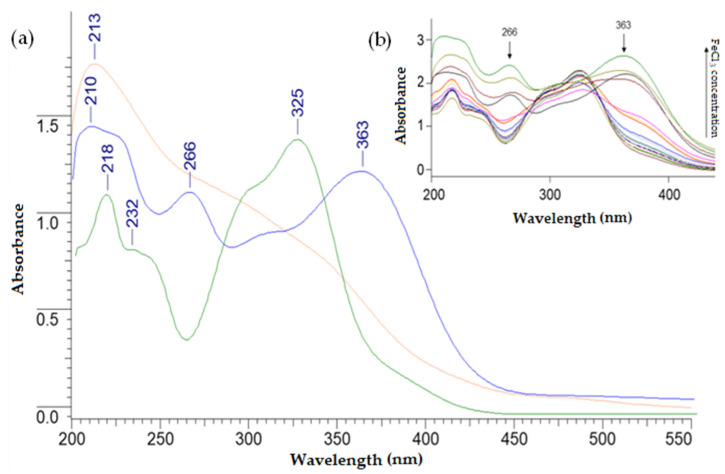
(**a**) UV/Vis spectra of FeCl_3_ in tris-HCl buffer, with C = 10^−4^ M (red line), 5-CQA in tris-HCl buffer, with C = 10^−4^ M (green line), and iron(III) with 5-CQA complex in 1:1 molar ratio in tris-HCl buffer, with C = 10^−4^ M (blue line) recorded in the range of 200–550 nm. (**b**) The UV/Vis spectra of successive series of prepared solutions; the increasing concentration of FeCl_3_ is depicted.

**Figure 6:**
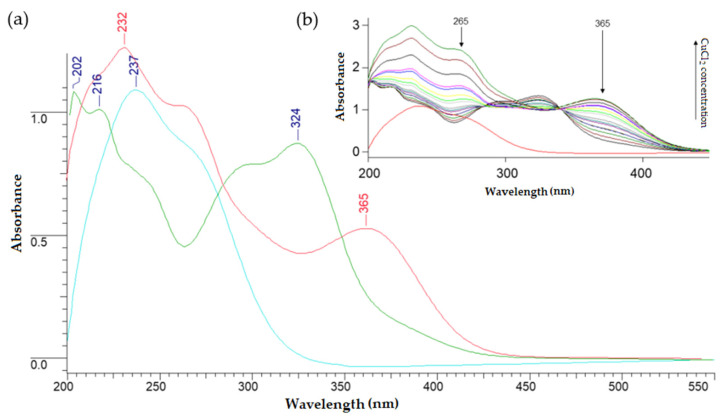
(**a**) The UV-Vis spectra of CuCl_2_ in tris-HCl buffer, with C = 10^−4^ M (blue line), 5-CQA in tris-HCl buffer, with C = 10^−4^ M (green line), and copper(II) with 5-CQA complex in 1:1 molar ratio in tris-HCl buffer, with C = 10^−4^ M (red line), recorded in the range of 200–550 nm. (**b**) The UV/Vis spectra of successive series of prepared solutions; the increasing concentration of CuCl_2_ is depicted (red line—CuCl_2_ spectrum in tris-HCl, with C = 10^−4^ M).

Figure 5 and Figure 6 show changes in the absorbance of the solutions due to the formation of Fe(III) and Cu(II) complexes with 5-CQA. In the UV/Vis spectrum of 5-CQA, four bands were present at about 218, 232, 298, and 325 nm. These bands arose from the π→π* transitions within the aromatic ring and the C=C double bond [10]. Due to the complex formation, the bands at 232 and 325 nm disappeared and new bands at ~266 and ~363 nm appeared. As the concentration of metal ions increased, the absorbance of the band at 218 nm increased and the absorbance of the band at 298 nm decreased. The isosbestic point in the absorption spectrum resulted from the formation of an iron(III) complex with chlorogenic acid in the solution. Figure 5 shows selected spectra of the successively prepared solutions. In the spectrum of 5-CQA, four bands were present at 218, 232, 298, and 325 nm. The bands at 298 and 218 nm were derived respectively from the n→π* and π→π* electronic transitions within the C=O group, whereas the bands at 232 and 325 were assigned to the π→π* transitions within the aromatic ring [10]. In the UV/Vis spectra of the Fe(III) and Cu(II) complexes with 5-CQA, the bands at 232 and 325 nm were shifted to ~265 and ~365 nm, which suggested that the metal complexation strongly affected the electronic charge distribution in the catechol moiety. This could have been caused by the participation of the –OH substituents from the aromatic ring in the metal ion coordination.

**Figure 7:**
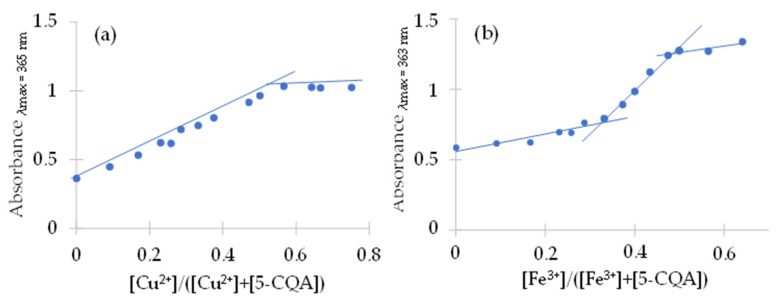
The mole-ratio plot for the estimation of the composition of (**a**) Cu(II) and (**b**) Fe(III) chlorogenate complexes.

The mole ratio is suitable for examining the composition of chlorogenic acid complexes with iron(III) and copper(II). As a result of the conducted research, the presence of complexes of chlorogenic acid with copper(II) in a molar ratio of 1:1 was found in an aqueous solution (Figure 7a). In the case of the iron(III) complex with chlorogenic acid (Figure 7b), we observed two pitches in the curve, indicating the coexistence of two forms of the complex in solution, in a molar ratio of 1:1 and 1:2 (metal:ligand). According to the literature, chlorogenic acid forms complexes with Cu(II), Mn(II), Zn(II), and Fe(III) and the formula of these compounds was estimated as ML_n_, where L is the chlorogenic acid and n = 1, 2, or 3 depending on the pH of the aqueous solution [51]. The authors claimed that in the pH range of 5–6.5, there was an equilibrium between the two forms of the complex (FeL/FeL_2_^3−^), and when pH ≤ 5, the neutral form existed (FeL). The higher complex, FeL_3_^6−^, was formed at a pH of ~7.7. In the case of the Cu(II) complex with chlorogenic acid, at a pH = 5.7, the main form was CuL^−^, whereas at a higher pH, the CuL_2_^4−^ complex occurred, which was the major species at pH = 7.3. Other studies revealed that at a nearly neutral pH, Cu(II), Fe(II), and Mn(II) were complexed by 5-CQA with a molar ratio of 1:1 [52]. According to Milic et al., for an aqueous solution at a pH of 7.5, the estimated stoichiometry was 1:1 for Pb(II) 5-CQA and 1:1 and 1:2 for Cu(II) 5-CQA [53]. Taking into account the pH of the solution, it is necessary to consider the participation in the coordination of the metal ion not only of the carboxylate group, but also of the hydroxylic groups of the catechol group [41], as well as the additional possible reduction of iron(III) ions to iron(II) and the formation of chlorogenic acid oxidation products [41]. Studies on the complex of 5-CQA with vanadium(IV) have also confirmed the participation of catechol groups in metal binding [18]. Therefore, the coordination of iron(III) and copper(II) ions through the catechol moiety should also be considered. On the basis of Figure 5 and Figure 6, the stability constants (logK) for the complexes were calculated. For Cu(II) 5-CQA, the stability constant was logK = 4.23, whereas for Fe(III) 5-CQA it was logK_1_ = 5.20 (metal:ligand 1:1) and logK_2_ = 4.56 (metal:ligand 1:2).

The difference in the composition of the complexes in solid form and in solution may be explained by a slightly different pH of the reaction environment. In solution, the pH was kept at 7.4 by the presence of the tris-HCl buffer, whereas during the synthesis of the complexes in solid form, the solution of NaOH was added to 5-CQA to deprotonate the acid and facilitate the formation of metal complexes (pH = ~8.2). The slightly higher pH probably resulted in an increase in the amount of the deprotonated form of 5-CQA with the participation of the –OH group at the para position of the catechol moiety. This might have caused the formation of the complexes in solid form with different molar stoichiometry (metal:ligand) compared to the complexes in solution. The stability constants for 5-CQA were: pKa1 (COOH) = 3.35; logKa2 (OH in the para position) = 8.30; and pKa3 (OH in the meta position) = 12.06 [51].

### 3.4. DPPH^•^, HO^•^, and ABTS^•+^ Antiradical Activity Assays

**Figure 8:**
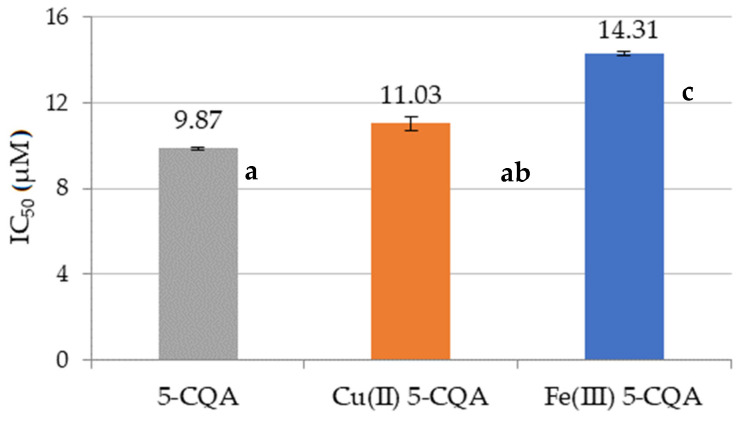
Antioxidant properties of 5-CQA, Cu(II) 5-CQA, and Fe(III) 5-CQA, expressed as the ability to scavenge DPPH^•^ radicals (IC_50_). Mean values from three independent experiments ± SDs are shown. The same letter near the means indicates no significant difference (Tukey’s test, *p* < 0.05).

The antioxidant properties of chlorogenic acid and its complexes with Fe(III) and Cu(II) ions were measured using a DPPH^•^ assay and expressed as the concentration of antioxidant required to inhibit 50% of the DPPH^•^ radicals (IC_50_) [45]. The obtained results are presented in Figure 8. The value of the IC_50_ parameter equaled 9.87 ± 0.05 μM for 5-CQA, 11.03 ± 0.34 μM for Cu(II) 5-CQA, and 14.31 ± 0.08 μM for Fe(III) 5-CQA. 5-CQA was found to have the greatest ability to scavenge DPPH^•^ radicals from the studied compounds. Similar IC_50_ values to those obtained in the DPPH^•^ assay for 5-CQA can be found in the literature. For example, in a study by Zheng et al. [54], the IC_50_ for 5-CQA was 6.9 ± 0.1 μM [54]. In other studies, chlorogenic acid inhibited 50% of the DPPH^•^ radicals at a concentration of 7.23 ± 0.76 [7] and 7.39 ± 0.71 μM [20]. It can be concluded that 5-CQA is an effective scavenger of DPPH^•^ radicals.

**Figure 9:**
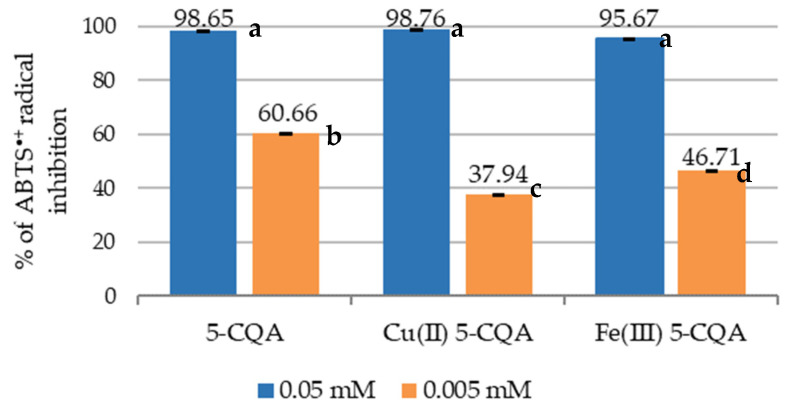
Antioxidant properties of 5-CQA, Cu(II) 5-CQA, and Fe(III) 5-CQA (0.05 and 0.005 mM) expressed as the ability to scavenge ABTS^•+^ cation radicals (%I). Mean values from three independent experiments ± SDs are shown. The same letter near the means indicates no significant difference (Tukey’s test, *p* < 0.05).

The ABTS^•+^ cation radical assay was carried out for two concentrations of the tested compounds (0.05 and 0.005 mM). The results are shown in Figure 9. The antioxidant activity increased with increasing compound concentration. All the studied compounds showed a significant radical scavenging ability (ranging from 95.67 to 98.76%) at a concentration of 0.05 mM. Larger differences in the activity of the tested compounds were observed at a concentration of 0.005 mM. The methanol solution of 5-CQA showed a higher ABTS^•+^ cation radical scavenging activity (60.66 ± 0.002%) than its complexes with Cu(II) and Fe(III) ions (37.94 ± 0.03% and 46.71 ± 0.01%, respectively).

**Figure 10:**
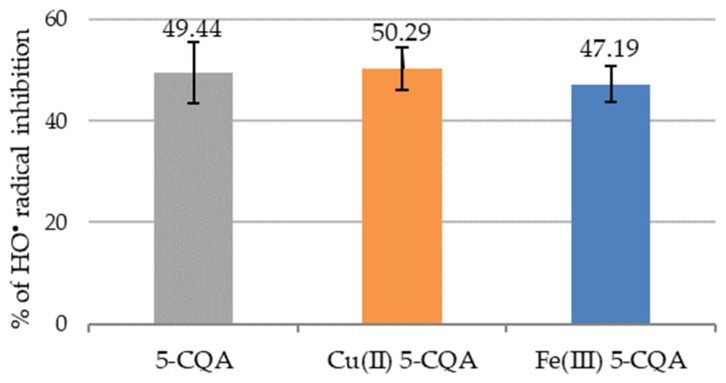
Antioxidant properties of 5-CQA, Cu(II) 5-CQA, and Fe(III) 5-CQA (0.1 mM) expressed as the ability to scavenge HO^•^ radicals (%I). Mean values from three independent experiments ± SDs are shown. There were no significant statistical differences between particular compounds (Tukey’s test, *p* < 0.05).

The hydroxyl radical (HO^•^) is a highly reactive oxygen species that is produced by the Fenton reaction [46]. The antioxidant activity of chlorogenic acid and the studied chlorogenates was measured as the ability of these compounds to scavenge HO^•^ radicals. The obtained results are presented in Figure 10. In this study, 5-CQA and Cu(II) 5-CQA showed a higher percentage of HO^•^ radical inhibition (49.44 ± 6.05% and 47.19 ± 3.56%, respectively) compared to Fe(III) chlorogenate (%I = 47.19 ± 3.56%). No significant differences were observed between the results obtained for the individual compounds.

The obtained results indicated that the complexation of chlorogenic acid with Cu(II) and Fe(III) ions did not significantly increase their antioxidant properties when measured in DPPH^•^, HO^•^, and ABTS^•+^ antiradical activity assays (for a sample concentration of 0.05 mM). The greatest differences in the results were obtained in the ABTS^•+^ cation radical assay for compounds at a concentration of 0.005 mM. In this assay, chlorogenic acid (0.005 mM) showed significantly higher radical-scavenging properties (60.66%) compared to its complexes with Fe(III) or Cu(II) ions (46.71 and 37.94%, respectively) (Figure 9). These three tests were based on mixed HAT (hydrogen atom transfer) or SET (single electron transfer) mechanisms of reaction, which more or less depend on the pH and the type of solvent. In the HAT mechanism, the bond dissociation energy is an important factor influencing the antioxidant effect, while in the SET reaction, such a parameter is the ionization potential [55,56]. In an acidic pH, the antioxidant undergoes protonation, which decreases the ionization potential and its ability to scavenge radicals, whereas in an alkaline pH, the proton dissociation increases, which facilitates the scavenging of the radicals [20]. The stability constants for 5-CQA were the following: pKa1 = 3.35, pKa2 = 8.30, and pKa3 = 12.06 [50]. Under acidic conditions, the protonated form (AH_3_) and the monoanion (AH_2_¯) were the main species, whereas in a neutral or basic pH, AH_2_¯ and the dianion AH^2^¯ were the dominant forms of 5-CQA. Above pH~11.2, the trianionic form A^3^¯ was dominant. Taking into account the experimental conditions in the DPPH, ABTS, and hydroxyl radical assays, the three species AH_3_, AH_2_¯, and AH^2^¯ should be considered. In the pH range of 4–8, the main form was AH_2_¯ (at pH > 7, the successive deprotonation starts, forming AH^2^¯), which is responsible for the antioxidant properties of 5-CQA in the ABTS and DPPH assays, although some authors have claimed that for the DPPH assay, the pH is irrelevant due to the use of an organic solvent (methanol, in our case) [57]. In the hydroxyl radical assay, the AH form should be predominant, which hinders the formation of complexes with metals. Therefore, there were no distinct differences in the hydroxyl radical assay in the antioxidant activity between 5-CQA and its metal complexes. In both the DPPH and ABTS assays, the antioxidant properties of 5-CQA were higher because Fe(III) and Cu(II) were coordinated through the carboxylate group, and at pH > 7, the deprotonation of the –OH of the aromatic ring enabled the additional metal coordination through the catechol moiety. The participation of the catechol moiety in the metal coordination decreased the radical scavenging properties of the metal complexes compared to the ligand alone.

There are some other reports in the literature concerning the antiradical activity of metal complexes with chlorogenic acid. In a study by Kalinowska et al. [7], a CQA/Zn(II) complex inhibited the DPPH^•^ and ABTS^•+^ radical scavenging activity more than chlorogenic acid alone. The values of the IC_50_ parameter in the DPPH^•^ assay were 5.45 and 7.23 µM for the CQA/Zn(II) and CQA, respectively. In the ABTS^•+^ assay, at a compound concentration of 25 µM, CQA/Zn(II) inhibited ABTS^•+^ cation radicals by 97.65%, while CQA inhibited the radicals by 89.53% [7]. Other work by Kalinowska et al. [20] showed that Li, Na, K, Rb, and Cs ions increased the antioxidant activity of the chlorogenic acid measured in DPPH^•^ and FRAP assays compared to that of the ligand alone, but with one exception. CQA/Li in a concentration of 5 μM showed slowly lower activity (106.92 µM Fe^2+^) than CQA in the same concentration (114.22 µM Fe^2+^) [20].

Moreover, in the literature there are many other examples of the complexation of phenolic compounds with the transition metals Fe(III) and Cu(II). Rutin, taxifolin, (-)-epicatechin, and luteolin complexes with Fe(III) and Cu(II) ions were synthesized in the work of Kostyuk et al. [58]. In their research, it was found that the obtained flavonoid complexes (flavonoid:metal ion ratio of 1:1) showed a significantly higher scavenging power than the free ligands. For example, in their tests, rutin alone inhibited the superoxide-driven reduction of NBT by 50% at a concentration of 9.0 μM, while the IC_50_ parameters for its complexes with Cu(II) and Fe(III) ions were 0.5 μM and 2.5 μM, respectively [58]. A study conducted by Dowling et al. [59] showed that Cu(II) genistein and biochinin A complexes (flavonoid:metal ion ratio of 2:1) exhibited greater antioxidant activity against DPPH^•^ radicals than free isoflavones, while the chelation of the same ligands with Fe(III) ions increased their pro-oxidant activity against ligands [59]. It can be concluded that the chelation of phenols does not always increase antioxidant ligand properties.

### 3.5. Inhibition of Linoleic Acid Peroxidation Assay

**Figure 11:**
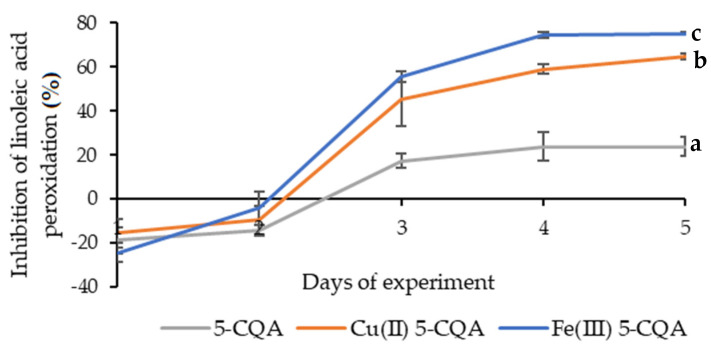
Linoleic acid peroxidation inhibition assay of 5-CQA, Cu(II) 5-CQA, and Fe(III) 5-CQA (0.001 M). Mean values from three independent experiments ± SDs are shown. The same letter near the means indicates no significant difference (Tukey’s test, *p* < 0.05).

The inhibitory activity of linoleic acid peroxidation by 5-CQA, Cu(II) 5-CQA, and Fe(III) 5-CQA was measured for 5 days. As shown in Figure 11, there were no significant differences in the inhibitory activity between the studied compounds on the first and second days of the experiment. An amount of 55.41 ± 2.40% inhibition by Fe(III) 5-CQA, 45.49 ± 12.50% inhibition by Cu(II) 5-CQA, and 17.38 ± 3.44% inhibition by 5-CQA were observed on the third day of measurement. The greatest differences in the activity of the compounds were observed on the fourth day of measurement, where the inhibition of linoleic acid peroxidation for Fe(III) 5-CQA was 74.60 ± 1.24%; for Cu(II) 5-CQA, it was 59.09 ± 2.36%; and for 5-CQA, it was 23.74 ± 6.61%. On the fifth day of the experiment, Fe(III) 5-CQA and Cu(II) 5-CQA inhibited the peroxidation of linoleic acid by 75.22 ± 0.47% and 64.87 ± 1.48%, respectively, while 5-CQA only inhibited it by 23.91 ± 4.20%. The results of this assay indicated that the synthesized chlorogenic acid complexes inhibited the peroxidation of linoleic acid more effectively than the ligand itself. This fact could be useful for designing compounds that are capable of extending the shelf life of oleaginous food.

### 3.6. Pro-Oxidant Activity Assay

**Figure 12:**
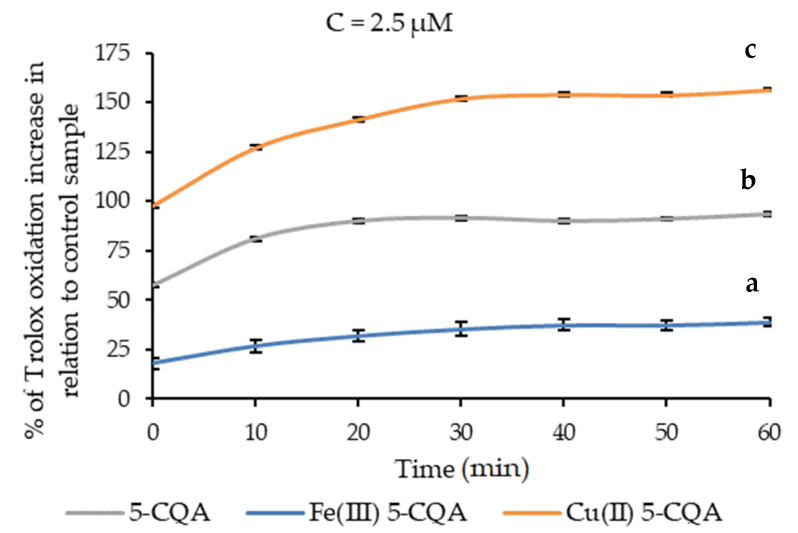

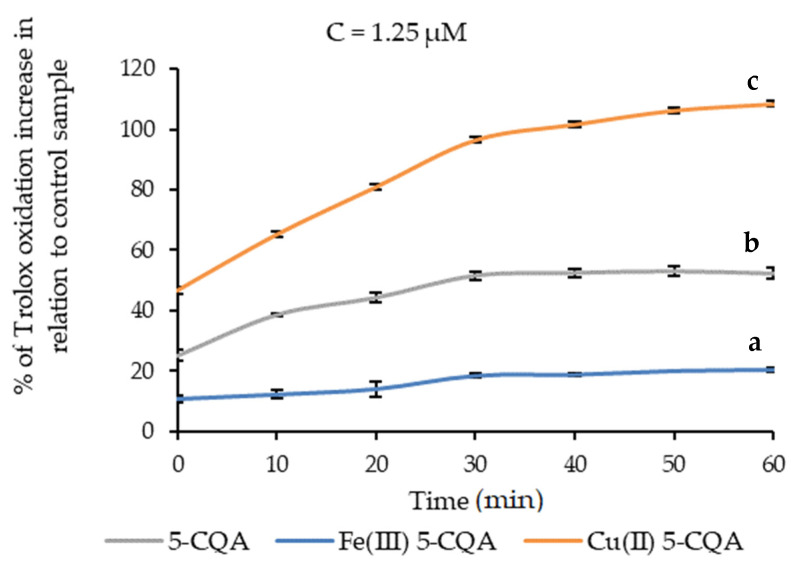
Pro-oxidation activity assay of 5-CQA, Cu(II) 5-CQA, and Fe(III) 5-CQA (2.5; 1.25 µM). Mean values from three independent experiments ± SDs are shown. The same letter near the means indicates no significant difference (Tukey’s test, *p* < 0.05).

The pro-oxidant activity of chlorogenic acid and its Cu(II) and Fe(III) complexes were measured for 60 min in two concentrations: 2.5 and 1.25 µM. As shown in Figure 12, the Cu(II) chlorogenate showed the strongest pro-oxidant activity and the Fe(III) chlorogenate had the lowest activity, while the chlorogenic acid itself had pro-oxidative power between these complexes. The pro-oxidant activity increased with time, with the highest values at 60 min in almost all cases. After 60 min, 5-CQA, Cu(II) 5-CQA, and Fe(III) 5-CQA at 1.25 and 2.5 µM concentrations increased the oxidation of Trolox by 108.46 ± 5.31% and 156.43 ± 3.58%; 52.23 ± 1.89% and 93.32 ± 13.99%; and 20.39 ± 0.81% and 38.81 ± 2.30%, respectively. The pro-oxidant activity of Cu(II) 5-CQA and Fe(III) 5-CQA was higher than that of the 5-CQA itself, which was also observed for the Zn 5-CQA complex in the work of Kalinowska and others [7].

When subjected to certain conditions (e.g., high concentration, occurrence of metal ions), some antioxidants can have pro-oxidant properties. One of the most well-known examples would be ascorbic acid, which displays a high pro-oxidant activity at a higher concentration. This is due to the overbalance of the reducing power over its antiradical activity. Low or moderately concentrated pro-oxidants can be beneficial for the defense system, but when they occur in excess, pro-oxidants cause oxidative imbalance [60]. It was also confirmed that in an oxygen environment, the presence of Cu or Fe can lead to the formation of reactive oxygen species [61]. Furthermore, this ability can be used in cancer therapy for inducing cancer cell apoptosis, for example through hydrogen peroxide generation [62].

### 3.7. Cell Viability Assay

**Figure 13:**
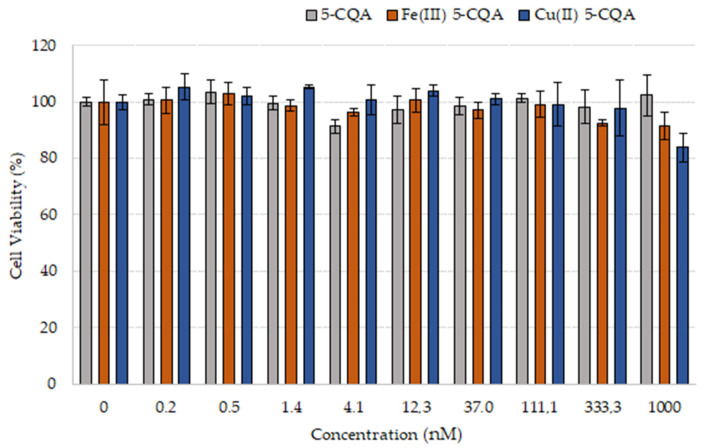
Viability of HaCaT cells after treatment with 5-CQA and its complexes with Fe(III) and Cu(II) (C = 0.15–1000 nM) for 24 h.

The influence of 5-CQA and the Fe(III) 5-CQA and Cu(II) 5-CQA complexes on the proliferation of HaCaT cells was investigated using an MTS assay (Figure 13). The obtained results indicated that 5-CQA alone does not influence the HaCaT cell viability, even in the wide concentration range of 0.15–1000 nM, and this result is comparable to other data obtained for various cell lines [54,55]. The Fe(III) 5-CQA and Cu(II) 5-CQA complexes were not toxic to cells in the tested concentration range either; however, at the highest applied concentration of 1000 nM, they reduced cell viability to 91.4 ± 4.9% and 83.9 ± 5.1%, respectively, whereas CQA alone did not influence HaCaT cell viability at this concentration (100.7 ± 4.5%). The one-way ANOVA of the viability of the HaCaT cells as a function of compound concentration for 5-CQA, Fe(III) 5-CQA, and Cu(II) 5-CQA at α = 0.05 revealed that there were no significant statistical differences in cell viability as a function of drug concentration for 5-CQA or Fe(III) 5-CQA. A statistically significant difference was found by ANOVA for the Cu(II) 5-CQA. A two-tailed unequal variance *t*-test at each drug concentration versus the control revealed that the only statistically significant difference was between the control and the highest concentration of the Cu(II) 5-CQA series. The higher reduction in cell viability caused by Cu(II) 5-CQA compared to Fe(III) 5-CQA and 5-CQA may be explained by the higher pro-oxidant properties of Cu(II) 5-CQA compared to the other studied compounds (Figure 12).

### 3.8. Lipophilicity Assay

The chromatographic lipophilicity parameters were collected in Table 3. Different stationary phases were selected because the mechanism of molecule separation involves different type of interactions, i.e., hydrophobic van der Waals interactions in the case of C18 and C8 alkyl-modified silica stationary phases, π→π* interactions when the PHE phenyl-modified silica phase is applied, or hydrogen bond formation between -CN groups (in the case of the CN cyano-bonded silica phase) and HO- groups from separate molecules. On the basis of the result, it may be concluded that the Cu(II) and Fe(III) 5-CQA complexes showed significantly lower lipophilicity than the ligand alone. The compounds can be ordered according to their decreasing lipophilicity as follows: 5-CQA→Cu(II) 5-CQA→Fe(III) 5-CQA (determined in the C18, CN, and PHE stationary phases). The differences in the lipophilicity and solubility between 5-CQA and its Cu(II) and Fe(III) complexes may explain their different cytotoxic and even antioxidant properties as well as membrane permeability.

## 4. Conclusions

The Cu(II) and Fe(III) complexes of chlorogenic acid (5-CQA) were synthesized and studied using UV/Vis and FT-IR. The anti- and pro-oxidant properties of the obtained complexes and 5-CQA were measured using various assays (DPPH^•^, HO^•^, ABTS^•+^, linoleic acid peroxidation inhibition, and Trolox pro-oxidation). 5-CQA and the metal complexes revealed a high antioxidant activity. The pH of the assays was an important factor determining the degree of ligand complexation by the copper and iron ions and the participation of carboxylate groups and –OH substituents from the aromatic ring in metal ion coordination. The coordination of Fe(III) and Cu(II) ions by the catechol moiety of 5-CQA decreased the radical scavenging properties of the complexes compared to those of the ligand alone. On the other hand, the Fe(III) and Cu(II) complexes with 5-CQA retained lipid peroxidation to a greater extent than 5-CQA. Special attention should be paid to Fe(III) 5-CQA, which exhibited a lower pro-oxidant activity in the Trolox assay compared to Cu(III) 5-CQA and 5-CQA. Moreover, 5-CQA alone and its complexes with Fe(III) and Cu(II) cations were not toxic to HaCaT cells in a tested concentration range of 0.15–1000 nM after a 24 h incubation time. However, for the Fe(III) 5-CQA and Cu(II) 5-CQA compounds, a slight decrease in cell viability was observed at the highest applied concentration. Further studies are needed to explore this influence and its potential mechanism.

## Figures and Tables

**Table 1:** Results of thermal decomposition of Cu(II) and Fe(III) 5-CQAs in air atmosphere.

Complex	T_1_/°C	T_endo_	Mass Loss/%		Anhydrous Form	T_2_/°C	T_exo_	Residue/%		Residue
			Found	Calc.				Found	Calc.	
Fe_2_(C_16_H_17_O_9_)_3_·8H_2_O	30–140	91	10.69	10.95	Fe_2_(C_16_H_17_O_9_)_3_		191	12.97	12.14	Fe_2_O_3_
						140–405	221			
							344			
							370			
Cu(C_16_H_17_O_9_)_2_·5H_2_O	30–150	88	10.37	10.46	Cu(C_16_H_17_O_9_)_2_		210	10.06	9.25	CuO
						150–465	246			
							270			
							321			

T_1_—temperature range of dehydration. T_2_—temperature range of degradation of anhydrous complexes to suitable oxides. T_endo_—peak top of endothermic effect. T_exo_—peak tops of exothermic effect.

**Table 2:** Elemental analysis of Cu(II) and Fe(III) complexes with chlorogenic acid.

Compound Formula	C%		H%		M% (Based on TG Profile)	
	Exp	Calc	Exp	Calc	Exp	Calc
Cu(C_16_H_17_O_9_)_2_·5H_2_O	44.08	44.64	5.09	5.12	8.03	7.38
Fe_2_(C_16_H_17_O_9_)_3_·8H_2_O	43.16	43.78	4.69	5.09	9.07	8.49

**Table 3:** Lipophilicity parameters determined by chromatographic methods (the logarithm of the retention factor, log*k*_w_) for the Cu(II) and Fe(III) chlorogenates and chlorogenic acid [20].

Compound	C18	C8	CN	PHE
	log*k*_w_			
Cu(II) 5-CQA	0.45	0.04	1.28	0.80
Fe(III) 5-CQA	0.23	0.23	0.36	0.67
5-CQA [20]	3.88	1.11	2.09	1.45

## Data Availability

The data presented in this study are available upon request from the corresponding author.

## Supplementary Materials

The following supporting information can be downloaded at: https://www.mdpi.com/article/10.3390/ma15196832/s1, Table S1: Sources of CQA with extraction and determination methods; Table S2: Sources of different CQA isomers; Table S3: The wavenumbers, intesities and assignment of selected bands from the FT-IR spectra of Cu(II) and Fe(III) 5-CQAs and 5-CQA acid [7]; the symbols denote: ν—stretching vibrations, δ—deforming in plane and oop—out of plane bending vibrations; s—strong, m—medium, w—week, v—very, sh—on the slope.
