# Supplementary material for: Fe(III) and Cu(II) Complexes of Chlorogenic Acid: Spectroscopic, Thermal, Anti-/Pro-Oxidant, and Cytotoxic Studies
Source: Materials (Basel). 2022 Oct 1;15(19):6832. doi: 10.3390/ma15196832 (PMC9572621; doi:10.3390/ma15196832)
Supplement: Supplementary file 1 [file materials-15-06832-s001.zip › materials-1841240-supplementary.pdf]

Supplementary materials

Fe(III) and Cu(II) complexes of chlorogenic acid: spectroscopic, thermal, anti-/pro-oxidant and cytotoxic studies

Monika Kalinowska [1,*], Kamila Gryko [1], Ewelina Gołębiewska [1], Grzegorz Świderski [1], Hanna Lewandowska [2], Marek Pruszyński [2,3], Małgorzata Zawadzka [1], Maciej Kozłowski [1], Justyna Sienkiewicz-Gromiuk [4], Włodzimierz Lewandowski [1]

1 Department of Chemistry, Biology and Biotechnology, Institute of Environmental Engineering and Energetics, Faculty of Civil Engineering and Environmental Sciences, Białystok University of Technology, Wiejska 45E Street, 15-351 Białystok, Poland

2 Institute of Nuclear Chemistry and Technology, 16 Dorodna Street, 03-195 Warsaw, Poland

3 NOMATEN Centre of Excellence, National Centre of Nuclear Research, 7 Andrzeja Soltana Street, 05-400 Otwock, Poland

4 Department of General and Coordination Chemistry and Crystallography, Institute of Chemical Sciences, Faculty of Chemistry, Maria Curie-Sklodowska University in Lublin, Maria Curie-Sklodowska Sq. 2, 20-031 Lublin, Poland

* Correspondence: m.kalinowska@pb.edu.pl

Table S1. Sources of CQA with extraction and determination methods.

Source	CQA amount [mg/g]	Analytical method: Extraction	CQA determination	Ref.
Potato pellets	0.106 ± 0.004	Heating at 40°C/30 min	HPLC	[30]
	0.102 ± 0.007	Maceration at RT/48 h		
	0.049 ± 0.003	Soxhlet		
	0.083 ± 0.001	Reflux		
	0.032 ± 0.004	Percolation		
	0.094 ± 0.005	Ultrasound		
Etlingera elatior fresh leaves	2.94 ± 0.53	Methanolic extraction and filtration	HPLC	[31]
Etlingera fulgens fresh leaves	2.19 ± 0.14			
Lonicera japonica fresh flowers	1.15 ± 0.16			
Ipomoea batatas fresh leaves	1.73 ± 0.13			
Apples		Ultrasonic extraction using methanol acetic acid solution.	Reverse-phase HPLC	[32]
Idared peel	0.0715			
Idared pulp	0.1169			
Elstar peel	0.165 ± 0.013			
Elstar pulp	0.029 ± 0.002			
Fuji peel	0.174 ± 0.039			
Fuji pulp	0.107 ± 0.009			
Golden Delicious peel	0.037–0.017			
Golden Delicious pulp	0.057–0.029			
Granny Smith peel	0.06–0.006			
Granny Smith pulp	0.071–0.028			
McIntosh peel	0.2345			
McIntosh pulp	0.1512			
Pinova peel	0.248 ± 0.019			
Pinova pulp	0.067 ± 0.002			
Green ground coffee	47	Extraction using rotatory shaker and isopropanol solution.	Folin-Ciocalteau assay (UV-Vis)	[33]
Regular coffee	3.95–1.32 (3-CQA)	Brewed coffee treated with Carrez reagents I, II and methanol, then filtered.	HPLC	[34]
	4.56–1.44 (4-CQA)			
	7.06–2.13 (5-CQA)			
Decaffeinated coffee	3.42–0.45 (3-CQA)			
	3.78–0.51 (4-CQA)			
	6.23–0.82 (5-CQA)			
Coffee brand		Half-dry extraction method		
Timor + Yellow Catuaí Hybrid	5.028 ± 0.20 (3-CQA)	Extraction using aqueous methanol solution and Carrez solutions.	HPLC	[35]
	6.663 ± 0.24 (4-CQA)			
	43.175 ± 1.02 (5-CQA)			
Timor + Red Cattura Hybrid	5.105 ± 0.52 (3-CQA)			
	6.803 ± 0.21 (4-CQA)			
	30.052 ± 0.65 (5-CQA)			
Yellow Bourbon	4.039 ± 0.41 (3-CQA)			
	5.826 ± 0.74 (4-CQA)			
	33.050 ± 0.74 (5-CQA)			
Red Catuaí	4.379 ± 0.41 (3-CQA)			
	6.058 ± 0.30 (4-CQA)			

	32.263 ± 0.41 (5-CQA)			
Rubi	4.459 ± 0.17 (3-CQA)			
	6.179 ± 0.25 (4-CQA)			
	31.915 ± 0.54 (5-CQA)			
Topázio	5.360 ± 0.15 (3-CQA)			
	6.894 ± 0.28 (4-CQA)			
	32.512 ± 0.35 (5-CQA)			
		Wet extraction method		
Timor + Yellow Catuaí Hybrid	5.404 ± 0.74 (3-CQA)	Extraction conducted on soeked cherries using aqueous methanol solution and Carrez solutions.	HPLC	[35]
	7.323 ± 0.20 (4-CQA)			
	48.451 ± 0.84 (5-CQA)			
Timor + Red Cattura Hybrid	5.063 ± 0.14 (3-CQA)			
	6.620 ± 0.31 (4-CQA)			
	35.278 ± 0.15 (5-CQA)			
Yellow Bourbon	5.015 ± 0.17 (3-CQA)			
	6.052 ± 0.16 (4-CQA)			
	34.102 ± 0.18 (5-CQA)			
Red Catuaí	5.281 ± 0.23 (3-CQA)			
	6.853 ± 0.24 (4-CQA)			
	32.568 ± 0.42 (5-CQA)			
Rubi	4.211 ± 0.22 (3-CQA)			
	5.718 ± 0.21 (4-CQA)			
	36.826 ± 0.39 (5-CQA)			
Topázio	3.840 ± 0.15 (3-CQA)			
	5.032 ± 0.17 (4-CQA)			
	31.628 ± 0.19 (5-CQA)			
Coffea arabica Mundo Novo green	6.672 ± 0.15 (3-CQA)	Methanol-based extraction proces and Carrez solutions.	HPLC-MS	[36]
	8.075 ± 0.13 (4-CQA)			
	36.112 ± 0.63 (5-CQA)			
Coffea arabica Mundo Novo roasted	10.966 ± 0.26 (3-CQA)			
	10.966 ± 0.26 (4-CQA)			
	27.367 ± 0.73 (5-CQA)			
Coffea arabica Catuaí Vermelho green	6.180 ± 0.02 (3-CQA)			
	7.709 ± 13.4 (4-CQA)			
	33.574 ± 0.55 (5-CQA)			
Coffea arabica Catuaí Vermelho roasted	10.301 ± 0.11 (3-CQA)			
	12.534 ± 0.20 (4-CQA)			
	24.519 ± 0.17 (5-CQA)			
Coffea robusta Conillon green	10.656 ± 0.10 (3-CQA)			
	12.772 ± 0.14 (4-CQA)			
	41.140 ± 0.40 (5-CQA)			
Coffea robusta Conillon roasted	13.084 ± 0.00 (3-CQA)			
	16.665 ± 0.16 (4-CQA)			
	31.755 ± 0.04 (5-CQA)			

Table S2. Sources of different CQA isomers.

**Isomers**	**Sources**	**Content**	**Ref.**
3-CQA, 4-CQA, 5-CQA, 3,4-diCQA, 3,5-diCQA, 4,5-diCQA	*Coffea canephora* (Robusta)<br>*Coffea arabica* (Arabica)	5-10% d.w. of green beans<br>20-675 mg 5-CQA from 200 ml of roasted, ground coffee infusion	[2,4,5]
mono- and diCQA	Tea	1599 mg/100 g d.m.	[5]
mono- and diCQA	Yerba mate	16-41 mg CQA per 200 cm^3 from roasted mate<br>480-520 mg of 5-CQA with traditional brewing methods	[4]
CQAs (mainly 5-CQA)	Pears	-	[2]
CQAs (mainly 5-CQA)	Apples	12-31 mg/100 mL of apple juice	[4,5,8]
CQAs (mainly 5-CQA)	Plums	0.5 mg/g	[5]
CQAs (mainly 5-CQA)	Cherries	0.4 mg/g	[5]
CQAs (mainly 5-CQA)	Blueberries	2 mg/g	[2,5]
CQAs (mainly 5-CQA)	Citrus fruits	-	[9]
5-CQA	*Sorbus domestica*	1500 mg/kg	[4]
CQAs	Tomatoes	4.22 mg/100 g f.w.	[5]
CQAs	Brassica vegetables (kale, cabbage, brussels sprouts)	-	[4]
CQAs	Celery vegetables (celery, carrots, cumin, coriander)	-	[4]
CQAs	Sweet potatoes	0,2 mg/g	[5]
CQAs	Potatoes	500-1200 mg/kg d.m.<br>2-5 g/kg in peel	[4]
CQAs	Turnips	0.083 mg/g	[5]
CQAs	Carrots	0.062 mg/g	[5]
CQAs	*Cynara scolymus*	-	[4]
CQAs	*Scorzonera hispanica* L.	-	[4]
CQAs	*Echinacea purpurea* L. Moench	-	[4]
CQAs	*Achillea millefolium* L.	-	[4]
CQAs	*Silybum marianum* L. Gaertner	-	[4]
CQAs	*Tussilago farfara* L.	-	[4]
CQAs	*Tanacetum vulgare* L.	-	[4]
CQAs	*Matricaria chamomilla* L	-	[4]
CQAs	Mugwort	-	[12]
CQAs	Honeysuckle	-	[13]

Table S3. The wavenumbers, intesities and assignment of selected bands from the FT-IR spectra of Cu(II) and Fe(III) 5-CQAs and 5-CQA acid [7]; the symbols denote: ν – stretching vibrations, δ – deforming in plane and oop – out of plane bending vibrations; s – strong, m – medium, w – week, v –very, sh – on the slope.

**5-CQA**		**Cu(II) 5-CQA**		**Fe(III) 5-CQA**		**Assignment**
IR	int.	IR	int.	IR	int.	
1725	s	-	-	-	-	$\nu(C{=}O)_{COOH\ group}$
1685	vs	1685	s	1685	m	$\nu(C{=}O)_{ester\ group}$
1638	s	-	-	-	-	$\nu(C{=}C)$
1600		1594	vs	1614, 1583	m, m	$\nu(COO^-)_{asym}$
1529	m	-	-	-	-	$\nu(CC)_{ar}$
1517	m	1515	m	1524	m	$\nu(CC)_{ar}$
		1493	m	1481	m	$\nu(CC)_{ar}$
1442	s	1446	m	1429	m	$\delta(COH)_{quinic\ ring}$
-		1364	m	1356	m	$\nu(COO^-)_{sym}$
1323	m	-	-	-	-	$\nu(CC)+\ \delta(CCH)+\delta(COH)_{quinic\ ring}$
1303	s	-	-	-	-	$\nu(CO)+\nu(CC)+\delta(CCH)$
1286	vs	1261	vs	1259	vs	$\nu(C\text{-}O)_{catechol\ group}+\delta(CH)_{ar}$
1220	m	-	-	-	-	$\nu(CC)+\nu(CH)+\delta(CCH)+\delta(COH)$
1201	s	-	-	-	-	$\delta(COH)_{quinic\ ring}$
1182	vs	1182	s	1181	s	$\nu(CC)+\nu(CH)+\delta(CCH)+\delta(COH)$
-	s	1156	m	1154	m	$\nu(CC)+\nu(CH)+\delta(CCH)+\delta(COH)$
1133	s	-	-	-	-	$\nu(C\text{-}O)_{COOH\ group}$
1112	s	1119	m	1119	s	$\nu(C\text{-}O)_{ester\ group}$
1087	s	1087	m	1085	s	$\nu(CC)+\nu(C\text{-}O)_{quinic\ ring}$
1073	w	-	-	-	-	$\nu(CC)_{quinic\ ring}+\delta(CH_{quinic\ ring}$
1036	m	1040	m	1039	m	$\nu(C_{quinic\ ring}\text{-}O_{in\ ester\ group)}+\delta(CH)_{quinic\ ring}$
1000	w	-	-	-	-	oop(HC-C=C)+oop(HC=CH)
970	m	977	m	977	m	$\delta(CH)_{quinic\ ring}+\delta(CH)_{quinic\ ring}+\nu(C\text{-}O)_{quinic\ ring}$
908	m	916	m	915	m	$\nu(CC)+\nu(C\text{-}O)_{quinic\ ring}+\delta(CC)+\delta(CH)$
853	m	852	m	852	m	$\delta(HC\text{-}CO)+oop(CH)_{ester\ group}$
818	s	-	-	-	-	$\delta(CC)_{arom.\ ring}$
-		813	s	813	s	$\delta(CC)_{arom.\ ring}+\delta(COO^-)$
596		615	m	612	m	$oop(COO^-)$
